# Cost-effectiveness of neoadjuvant pembrolizumab plus chemotherapy with adjuvant pembrolizumab for early-stage non-small cell lung cancer in the United States

**DOI:** 10.3389/fimmu.2023.1268070

**Published:** 2023-09-26

**Authors:** Wentao Tian, Lishui Niu, Ziqi Wang, Ruoyu Lu, Gang Xiao, Fuxing Deng, Guilong Tanzhu, Rongrong Zhou

**Affiliations:** ^1^ Department of Oncology, Xiangya Hospital, Central South University, Changsha, Hunan, China; ^2^ Xiangya Lung Cancer Center, Xiangya Hospital, Central South University, Changsha, China; ^3^ National Clinical Research Center for Geriatric Disorders, Xiangya Hospital, Central South University, Changsha, China

**Keywords:** neoadjuvant, adjuvant, pembrolizumab, cost-effectiveness, early-stage, non-small cell lung cancer

## Abstract

**Introduction:**

Perioperative (neoadjuvant and adjuvant) pembrolizumab has shown favorable efficacy in patients with early-stage non-small cell lung cancer (NSCLC). This study aims to evaluate the cost-effectiveness of this treatment from the perspective of the United States healthcare payers.

**Methods:**

We established a Markov model to compare the cost-effectiveness of perioperative pembrolizumab with that of neoadjuvant chemotherapy in 21-day cycles, utilizing data from the phase 3 KEYNOTE-671 trial. Additional data were extracted from other publications or online sources. Sensitivity analyses were conducted to evaluate the robustness of the findings. A willingness-to-pay threshold of $150,000 per quality-adjusted life-years (QALYs) gained was established. The main outcomes of this study were the measurement of QALYs, overall costs, incremental cost-effectiveness ratio (ICER), and net monetary benefit (NMB).

**Results:**

During a 10-year time horizon, the total costs of perioperative pembrolizumab and the control treatment were $224,779.1 and $110,026.3, respectively. The QALYs were 4.19 and 2.97 for the two treatments, respectively, which led to an ICER of $94,222.29 per QALY gained. The NMB at the WTP threshold at $150,000 per QALY gained was $67,931.3. One-way sensitivity analysis identified the cost of pembrolizumab as the primary factor influencing cost-effectiveness. Probabilistic sensitivity analysis indicated a 97.7% probability of perioperative pembrolizumab being cost-effective at the WTP threshold.

**Conclusions:**

From the perspective of the United States healthcare payers, perioperative pembrolizumab is a cost-effective treatment for patients with early-stage NSCLC.

## 1 Introduction

Currently, surgical resection remains the primary treatment approach for patients diagnosed with early-stage non-small cell lung cancer (NSCLC) (1). However, surgery alone often fails to provide a complete cure for early-stage NSCLC, as there is a significant risk of local and distant recurrence following radical surgery, primarily attributed to occult micro-metastases (2, 3). Therefore, perioperative management and medications are currently one of the most frequently discussed issues.

Perioperative management includes neoadjuvant and adjuvant treatment. The National Comprehensive Cancer Network (NCCN) guidelines recommend adjuvant chemotherapy after surgery for patients with resectable stage IB-IIIA NSCLC, while neoadjuvant chemotherapy should also be considered for these patients (4, 5). Nevertheless, the efficacy of chemotherapeutic regimens seems limited, and exploring new and more effective neoadjuvant treatment regimens for early-stage NSCLC is pressing (6). Given the encouraging efficacy observed with immune checkpoint inhibitors (ICIs) either as monotherapy or in combination with chemotherapy for metastatic NSCLC, numerous studies are investigating the potential of ICIs in the adjuvant setting for surgically resected patients. Notably, based on the outcomes of the Impower010 trial, atezolizumab has emerged as the first ICI to receive approval from the United States Food and Drug Administration (FDA) for use as an adjuvant therapy in stage II-IIIA resectable NSCLC patients with a PD-L1 expression level of ≥1% (7). Additionally, treatments with ICIs alone or combined with chemotherapy have been investigated for the neoadjuvant treatment of NSCLC in recent years. Some studies showed that neoadjuvant ICIs could lead to immune activation and enhance the activity of tumor-antigen-specific T cells in the tumor microenvironment (8, 9), which may contribute to improving survival outcomes (9).

Several studies have assessed the administrations of ICIs in neoadjuvant or adjuvant settings for early-stage NSCLC. Checkmate 159, the first published pilot study on neoadjuvant ICI in NSCLC, evaluated the efficacy of preoperative nivolumab in 20 patients and showed that 9 patients had major pathological responses (MPR) and 2 patients had pathological complete responses (pCR) (10). The single-arm phase II LCMC3 trial, with a larger sample size (181 patients) showed that neoadjuvant atezolizumab and adjuvant chemotherapy revealed an MPR rate of 20% (29 of 143) and a pCR rate of 6% (8 of 143) (11). Apart from the above, neoadjuvant sintilimab and durvalumab also showed favorable efficacy (12, 13). Additionally, according to the phase III Checkmate 816 study on 358 patients, neoadjuvant nivolumab plus chemotherapy led to significantly higher pathologic responses (24% vs 2.2%, odds ratio [OR] 13.94, 99% confidence intervals [CI] 3.49-55.75; P<0.0001) compared with chemotherapy alone (14). Recently, the phase III KEYNOTE-671 trial compared perioperative pembrolizumab (neoadjuvant pembrolizumab + chemotherapy and adjuvant pembrolizumab monotherapy) with neoadjuvant chemotherapy alone in 797 patients with early-stage NSCLC. The results showed that the pembrolizumab group had superior event-free survival (EFS) compared to the chemotherapy alone group (62.4% vs 40.6%; hazard ratio [HR] = 0.58; 95% CI: 0.46-0.72; P<0.001) (15). Additionally, the pembrolizumab group demonstrated a higher pCR rate (18.1% vs 4.0%; 95% CI: 10.1-18.7; P<0.0001).

Although neoadjuvant treatment with ICIs offers several advantages, including the elimination of micrometastatic disease, improvement in the rate of complete resection, assessment of treatment response during surgical resection, and potential downstaging of tumors in some cases, it is essential to carefully consider the balance between these potential benefits, safety considerations, and cost-effectiveness when determining the most appropriate treatment for patients (6). Currently, a number of published studies have primarily examined the cost-effectiveness of ICIs for advanced non-small cell lung cancer (NSCLC), with only a limited number of analyses focusing on the cost-effectiveness of perioperative ICIs for early-stage NSCLC (16, 17). Specifically, cost-effectiveness studies for early-stage NSCLC are currently restricted to the evaluation of adjuvant atezolizumab treatment (18, 19). According to the findings reported in these publications, ICIs may demonstrate cost-effectiveness as a first-line treatment option for advanced NSCLC and as adjunctive therapy for early-stage NSCLC (18, 19). In this study, we evaluated the cost-effectiveness of perioperative (neoadjuvant and adjuvant) treatment with pembrolizumab in the US setting based on the results from the KEYNOTE-671 trial.

## 2 Materials and methods

This study followed the Consolidated Health Economic Evaluation Reporting Standards 2022 (CHEERS 2022), of which the checklist is displayed in the supplement.

### 2.1 Model structure

**Figure 1:**
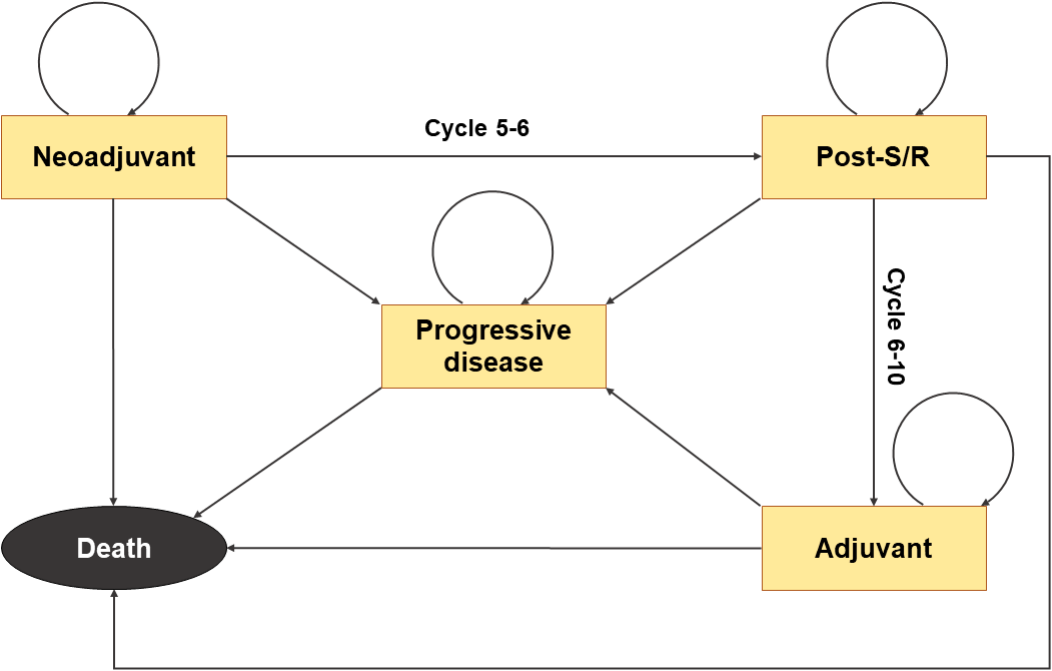
Structure of the Markov model. Death represents the absorbing state. The transformation from the neoadjuvant treatment state to the post-surgery/radiotherapy state is open only in cycles 5-6, and the transition from the post-surgery/radiotherapy state to the adjuvant treatment state is only possible during cycles 6-10.

A Markov model with 21-day cycles (**Figure 1**) was developed using the “heemod” package to estimate the health outcomes and cost-effectiveness of perioperative therapeutic strategies in early-stage NSCLC from the perspective of the United States healthcare payers (20). The KEYNOTE-671 trial included 797 adult patients with previously untreated, pathologically confirmed, stage II-IIIB NSCLC worldwide from over 20 countries (15). The patients were randomized in a 1:1 ratio to receive neoadjuvant pembrolizumab or placebo plus chemotherapy and adjuvant pembrolizumab or placebo. In the model, we set the analytic time horizon to 10 years because the therapeutic effects of the two treatments were assumed to be the same after 10 years from the initiation of therapy. Five states, including neoadjuvant treatment, post-surgery/radiotherapy, adjuvant treatment, progressive disease, and death, are involved in this model, with death being the absorbing state. The transformation from the neoadjuvant treatment state to the post-surgery/radiotherapy state and the transformation from the post-surgery/radiotherapy state to the adjuvant treatment state was only open in cycles 5-6 and cycles 6-10, respectively, resembling the design of the KEYNOTE-671 trial. The probabilities of transitions from other states to the progression or death state were estimated based on the EFS and OS data from the trial and/or the natural modality rate automatically obtained from the “heemod” package (20), all of which were time-varying.

The quality-adjusted life-years (QALYs), overall costs, incremental cost-effectiveness ratios (ICERs), and net monetary benefit (NMB) at a willingness-to-pay (WTP) threshold of $150000 per QALY gained were regarded as the primary outcomes (21). To discount both health outcomes and costs, a 3% discount rate per year was applied (22).

### 2.2 Clinical data

Data points of EFS and OS Kaplan-Meier curves from the KEYNOTE-671 trial were extracted using the GetData Graph Digitizer (version 2.26). Individual patient data were reconstructed using the “IPDfromKM” package (23), and Kaplan-Meier curves were recreated to ensure accuracy (**Figure S1** in the supplement). Long-term EFS and OS survival curves were fitted using the exponential, Weibull, log-logistic, log-normal, generalized gamma, gamma, and Gompertz distributions with the “flexsurv” package (**Figure S2** in the supplement). Subsequently, according to the Akaike Information Criterion (AIC) and Bayesian Information Criterion (BIC) values combined with visual inspections, the best-fit distribution models were chosen (**Table S1** in the supplement). Other clinical data, such as proportions of drug discontinuation, occurrence of severe adverse events (SAEs), number of patients who had surgery or radiotherapy, and number of patients who underwent neoadjuvant/adjuvant treatments, were also extracted (**Table 1**).

**Table 1:** Parameters input to the model.

Parameters	Base Value	Range		Distribution	Source
		Minimum	Maximum		
Clinical					
Body weight	70	52.5	87.5	Normal	Pei R, et al, 2021 (24)
BSA	1.86	1.40	2.33	Normal	Pei R, et al, 2021 (24)
Discount rate	0.03	0	0.08	Uniform	Kohn CG, et al, 2017 (25)
Log-normal OS survival model of the Pem	meanlog: 5.023	ND	ND	ND	Model fitting
	sdlog: 2.074				
Weibull OS survival model of Placebo	shape: 1.232	ND	ND	ND	Model fitting
	scale: 70.275				
Generalized gamma EFS survival model of Pem	mu: 2.866	ND	ND	ND	Model fitting
	sigma: 1.951				
	Q: -1.399				
Generalized gamma EFS survival model of Placebo	mu: 2.677	ND	ND	ND	Model fitting
	sigma: 1.316				
	Q: -0.498				
30-day post-surgery mortality*	0.023	0.017	0.029	Beta	Iniguez CEB, 2016 (26)
Treatment cost, $					
Pembrolizumab (per mg)	54.81	41.11	68.51	Gamma	Medicare drug prices (27)
Cisplatin (per mg)	0.32	0.24	0.40	Gamma	Medicare drug prices (27)
Pemetrexed (per mg)	6.45	4.84	8.06	Gamma	Medicare drug prices (27)
Gemcitabine (per mg)	0.02	0.01	0.03	Gamma	Medicare drug prices (27)
Radiotherapy per event	16335.11	12251.33	20418.89	Gamma	Wolff HB, et al, 2020 (28)
Surgery per event	15687.42	11765.57	19609.28	Gamma	Chen D, et al, 2021 (29)
Other costs, $					
Chemotherapy infusion					
First 1 hour	132.16	99.12	165.2	Gamma	CMS (CPT 96413) (30)
Additional 1 hour	28.47	21.35	35.59	Gamma	CMS (CPT 96415) (30)
Subsequent infusion per hour	65.06	48.80	81.33	Gamma	CMS (CPT 96417) (30)
Best supportive care	3674.65	2755.99	4593.31	Gamma	Criss SD, et al, 2019 (31)
End of life	17909.24	13431.93	22386.55	Gamma	Aguiar PN Jr, et al, 2018 (32)
Follow up	545.92	409.44	682.40	Gamma	Klein R, et al, 2010 (33)
SAE management cost (per event), $					
Anemia	2024.32	1518.24	2530.40	Gamma	Insinga RP, et al, 2018 (17)
Neutrophil count decreased	1276.52	957.39	1595.65	Gamma	Insinga RP, et al, 2018 (17)
Platelet count decreased	2220.14	1665.11	2775.18	Gamma	Insinga RP, et al, 2018 (17)
Utility					
Radiotherapy	0.79	0.71	0.87	Beta	Wolff HB, et al, 2020 (28)
Surgery	0.73	0.66	0.80	Beta	Wolff HB, et al, 2020 (28)
PD	0.65	0.59	0.72	Beta	Wolff HB, et al, 2020 (28)
PF	0.75	0.68	0.83	Beta	Wolff HB, et al, 2020 (28)
Disutility					
Anemia	0.25	0.23	0.28	Beta	Nafees B, et al, 2017 (34)
Neutropenia	0.07	0.06	0.08	Beta	Wan X, et al, 2019 (35)
Thrombocytopenia	0.35	0.32	0.39	Beta	Nafees B, et al, 2017 (34)
Risk of SAEs in the Pem group					
Anemia	0.07	0.05	0.09	Beta	H. Wakelee, et al, 2023 (15)
Neutropenia	0.21	0.16	0.26	Beta	H. Wakelee, et al, 2023 (15)
Thrombocytopenia	0.05	0.04	0.06	Beta	H. Wakelee, et al, 2023 (15)
Risk of SAEs in the Placebo group					
Anemia	0.06	0.05	0.08	Beta	H. Wakelee, et al, 2023 (15)
Neutropenia	0.20	0.15	0.25	Beta	H. Wakelee, et al, 2023 (15)
Thrombocytopenia	0.06	0.05	0.08	Beta	H. Wakelee, et al, 2023 (15)

*Mortality of radiotherapy is ignored due to a rather small proportion of patients undertaking radiotherapy.

OS, overall survival; EFS, event-free survival; ND, not determined; CMS, Centers for Medicare & Medicaid Services; HR, hazard ratio; SAE, severe adverse event; Pem, pembrolizumab; ND, not determined.

### 2.3 Cost and utility data

In this study, we focus on cost in the United States and effect differences in patients’ therapeutic phase. Therefore, we assumed that the two groups of patients had equivalent costs for diagnosis, including expenses for biochemical testing, pathological examination, and venipuncture-related protocols, as well as costs for treatment of recurrence and rehabilitation. We considered only the direct medical costs, which encompassed expenses related to drug usage, surgery, radiotherapy, intravenous infusion, routine follow-up, end-of-life care, best supportive care, and management of SAEs (**Table 1**). Based on the design of the KEYNOTE-671 trial, patients in the pembrolizumab arm receive neoadjuvant pembrolizumab (200 mg) combined with cisplatin-based chemotherapy every 21 days for 4 cycles, followed by surgery and (or) radiotherapy and adjuvant pembrolizumab (200 mg) triweekly for up to 13 cycles. The placebo arm received a placebo plus cisplatin-based chemotherapy as neoadjuvant therapy followed by surgery and (or) radiotherapy and an adjuvant placebo. Following disease progression, we assumed patients would receive radiotherapy, platinum-based chemotherapy for 6 cycles, and the best supportive care until death, according to the latest NCCN guideline (5). To estimate the dose of agents, we assumed a typical 65-year-old patient with 70 kg in weight and 1.86 m^2^ in body surface area (BSA) (36). All values of costs were collected from the Centers for Medicare & Medicaid Services and published articles (17, 27–33) and inflation-adjusted to 2023. The utility-scale was from 0 (death) to 1 (perfect health), and different utilities represented particular health states. We refer to previously published articles and obtained utilities associated with survival and health state and disutilities related to SAEs (28, 34) (**Table 1**).

### 2.4 Sensitivity analysis

To assess the model’s robustness, one-way sensitivity analysis and probabilistic sensitivity analysis (PSA) were adopted. In the one-way sensitivity analysis, a variance of ± 25% to cost values, proportions, and BSA was set as the upper and lower limits, and a variance of ±10% was set to the utility values. The upper and lower limits of the discount rate were set to 0.08 and 0, respectively. In the PSA, probabilistic distributions were assigned to costs (gamma distribution), proportions (beta distribution), utility values (beta distribution), BSA (normal distribution), and discount rates (uniform distribution). The mean value and standard deviation of the distributions, if applicable, were set to the baseline values and 10% of the baseline values, respectively. PSA was performed through 1,000 Monte Carlo repetitions across all distributions.

Additionally, several sets of scenario analyses were adopted. In the first set, we changed the time horizon to 5 years, 15 years, and 20 years, respectively, to estimate the cost-effectiveness of various patients’ life expectancies. In the second set, we reasonably chose different distributions for patients’ survival curves to evaluate the impact of choosing distinct distributions between two groups on cost-effectiveness. Furthermore, different prices of pemetrexed or pembrolizumab were adopted to test the robustness of the results. All the analyses are conducted using the R software (version 4.2.1).

## 3 Results

### 3.1 Base case results

The Markov model closely imitated and reasonably predicted the 10-year survival and treatment status of patients enrolled in Keynote-671 (**Figure S3**).

For the base case scenario (**Table 2**), the mean 10-year cost of perioperative pembrolizumab and placebo was $224,779.1 and $110026.3 per patient, respectively, and the QALY of patients in the 2 arms was 4.19 and 2.97, respectively. The incremental cost and QALY of the pembrolizumab arm were $114,752.8 and 1.217894 compared with those of the placebo arm, respectively, which yielded an ICER of $94,222.29 per QALY gained compared with the control. At a WTP threshold of $150,000 per QALY gained, the NMB of perioperative pembrolizumab was $67,931.3 compared with the control arm.

**Table 2:** Base case results.

Treatment	Cost, $	Incremental Cost, $	QALY	Incremental QALY	NMB*	ICER ($/QALY)
Pembrolizumab	224779.1	114752.8	4.19	1.22	67931.3	94222.29
Placebo	110026.3	NA	2.97	NA	NA	NA

*At a willing-to-pay threshold of $150,000 per QALY gained.

QALY, quality-adjusted life year; NMB, net monetary benefit; ICER, incremental cost-effectiveness ratio; NA, not applicable.

### 3.2 Sensitivity analyses

**Figure 2:**
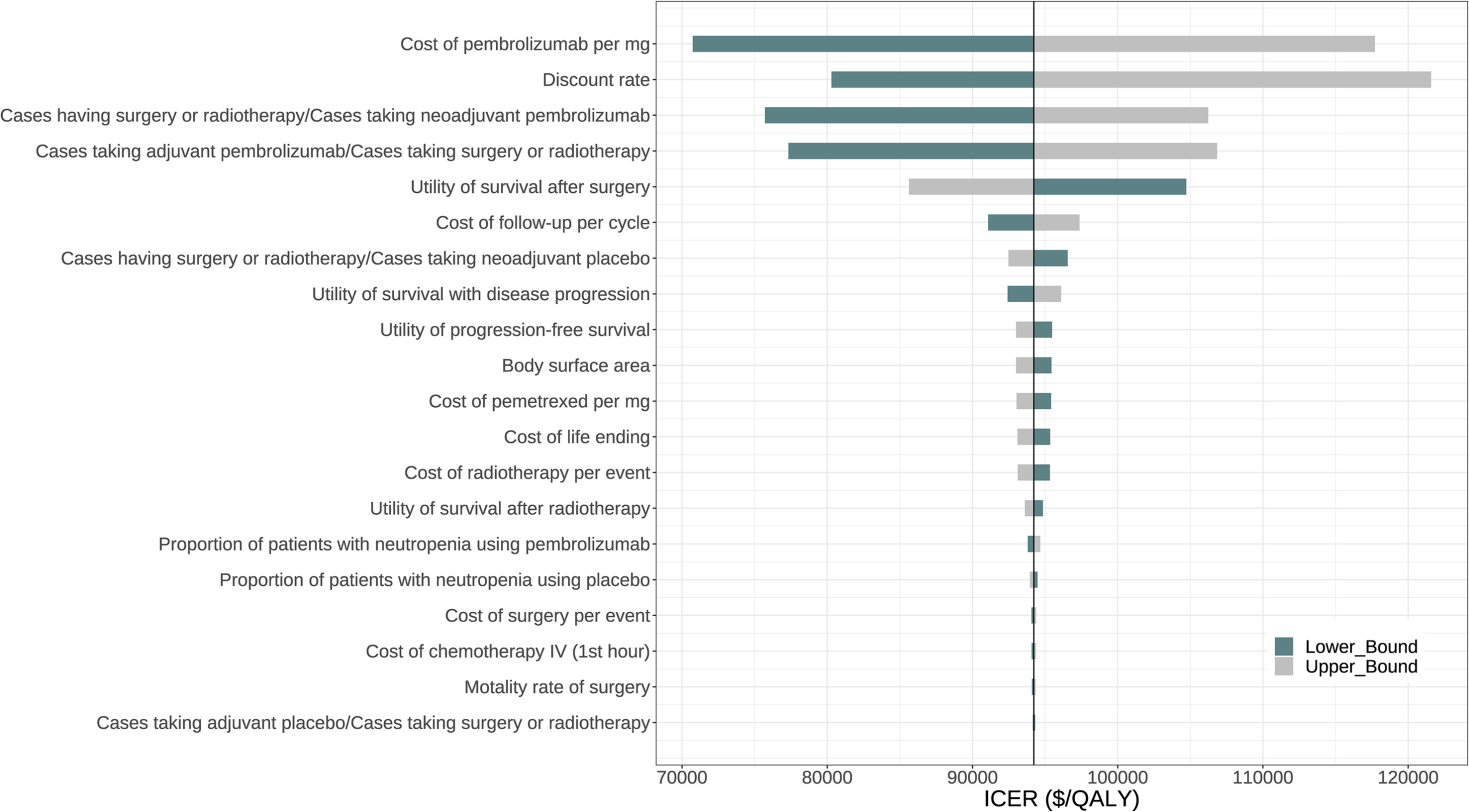
One-way sensitivity analysis.

**Figure 2** displays the tornado diagram, illustrating the 20 most influential parameters in the one-way sensitivity analyses. The complete tornado diagram, encompassing all parameters, is provided in **Figure S4**. The results demonstrated that the ICER of perioperative pembrolizumab compared to placebo was highly sensitive to the price of pembrolizumab per mg. When the lower boundary ($41.1075) and upper boundary ($68.5125) of this parameter were considered, the ICER ranged from $70,736.68 to $117,707.90 per QALY gained. Other influential parameters included the discount rate, the proportion of patients who underwent surgery or radiotherapy among those receiving neoadjuvant pembrolizumab, the proportion of patients who received adjuvant pembrolizumab among those who underwent surgery or radiotherapy, and the utility value for survival after surgery (**Figure 2**).

**Figure 3:**
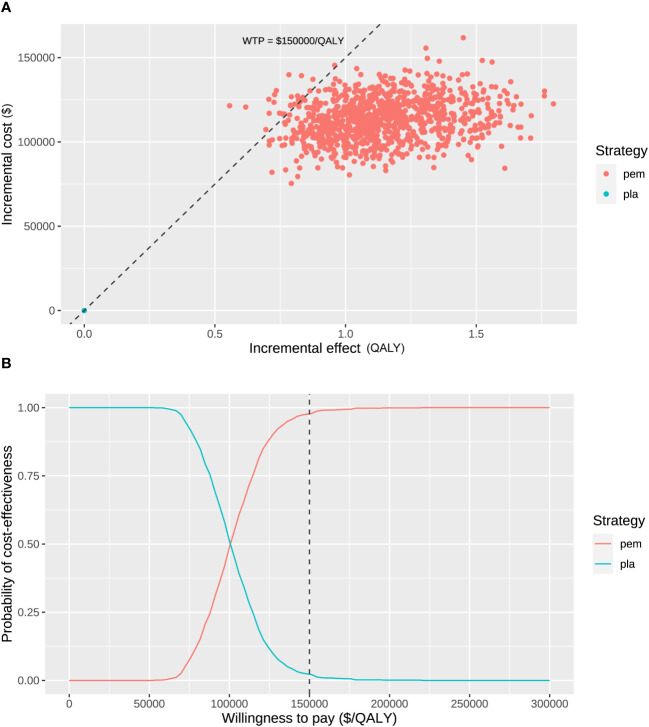
Probabilistic sensitivity analysis. **(A)** Incremental cost ($) and incremental effect (QALY) incurred by 1,000 probabilistic resamplings. **(B)** Probability of cost-effectiveness at varying willingness-to-pay. The dashed line represents the willing-to-pay threshold of $150,000 per QALY gained. *pem, pembrolizumab; pla, placebo*.

The probabilistic sensitivity analysis resampling 1,000 patients demonstrated that the total cost ranged from $170,816 to $275,049 for the pembrolizumab treatment and ranged from $83,864 to $133,904 for the placebo. The range of QALYs was 2.83 to 5.45 for pembrolizumab and 2.08 to 3.72 for the placebo control. Consequently, all samples yielded positive ICERs ranging from $52,461 to $218,361 per QALY gained. At a WTP threshold of $150,000 per QALY gained, the probability of perioperative pembrolizumab being a cost-effective strategy was 97.7% (**Figure 3**).

### 3.3 Scenario analyses

The supplementary **Table S2** showed the results of scenario analyses. First, we applied various time horizons to the model. By cutting down the time horizon to 5 years, the ICER increased to $315,842.3 per QALY gained, which was higher than the WTP threshold of $150,000 per QALY gained. On the contrary, by extending the time horizon to 15 years and 20 years, the ICER decreased to $63,019.22 per QALY gained and $53,379.72 per QALY gained. Second, because we used different distributions for OS estimation in the two arms (log-normal and Weibull distribution) in the base case analysis, we tried to unify the distributions to either the log-normal distribution or the Weibull distribution in the scenario analyses. When applying the Weibull distribution for both estimations, the ICER was $106,311.4 per QALY gained, and when the log-normal distribution was used, the ICER was $141,379.8 per QALY gained. Both of the ICERs were below the WTP threshold. Third, because we used the brand-name price of pemetrexed ($6.4 per mg) for the base case analysis but its actual price varied from $0.79 per mg (unnamed) to $1.23 per mg (Teva) across formulations, an additional scenario analysis was adopted using an average price of the cost of these formulations ($1.01 per mg) with the minimum and maximum as the lower and upper boundaries in the sensitivity analyses. Accordingly, the ICER was $98,226.31 per QALY gained (**Table S2**) and the corresponding sensitivity analyses supported the robustness of the result with a possibility of 97.2% of perioperative pembrolizumab being cost-effective at the WTP threshold (**Figure S5**). The results showed that perioperative pembrolizumab was still cost-effective when using pemetrexed at varied prices. Finally, as the result was primarily sensitive to the cost of pembrolizumab, we further assessed the cost-effectiveness assuming that the price of pembrolizumab was $65, $75, $85, and $95 per mg, respectively, which revealed ICERs of $111,687.6, $128,827.3, $145,966.9, and $163,106.6 per QALY gained. The result suggested that perioperative pembrolizumab might not be cost-effective if the price of it exceeded $85 per mg.

## 4 Discussions

Neoadjuvant and adjuvant immunotherapies have been widely studied and emerged as potential therapeutic strategies for early-stage NSCLC (37). Recently published results of the KEYNOTE-671 trial showed favorable efficacy and safety profiles of applying pembrolizumab in neoadjuvant and adjuvant settings for patients with early-stage NSCLC (15). However, the great expense of this treatment may form an obstacle to its wide application. According to the results of this study, from the perspective of the United States healthcare payers, perioperative pembrolizumab demonstrated superior cost-effectiveness compared with the placebo group. The sensitivity analyses and scenario analyses support the robustness of this finding.

Although both neoadjuvant and adjuvant treatments can reduce recurrence rates and extend patients’ survival, they aim differently in cancer management. Generally, the main goal of neoadjuvant treatments is to reduce tumor size before resection, while that of adjuvant treatments is to eliminate remaining cancer cells to prevent recurrence (6). As supported by a meta-analysis, neoadjuvant chemotherapy and adjuvant chemotherapy led to similar disease-free survival and OS in patients with early-stage NSCLC, while the neoadjuvant group was more likely to receive full doses and cycles of chemotherapy (P = 0.014/0.005) and had fewer SAEs (P=0.001) (38, 39). Accordingly, neoadjuvant treatments might decrease the costs for toxicity management and increase patients’ utilities compared to adjuvant therapies with the same agents. Specifically, several studies have investigated the cost-effectiveness of adjuvant ICI therapies for early-stage NSCLC. Based on the results of the Impower-010 study, the study by Das et al. (18) and the study by Chen et al. (40) showed that adjuvant atezolizumab was a cost-effective treatment for patients with early-stage NSCLC and PD-L1 expression ≥1% in the United States and China, respectively. Additionally, the studies conducted by Yip et al. (41) and Escudero-Vilaplana et al. (19) indicated that adjuvant atezolizumab was also cost-effective for early-stage NSCLC patients with PD-L1 expression ≥50% in the United Kingdom and Spain. However, despite the positive findings on the cost-effectiveness of adjuvant ICI therapy in early-stage NSCLC, there is a lack of studies exploring the cost-effectiveness of neoadjuvant and perioperative (neoadjuvant and adjuvant) ICI treatments.

As for pembrolizumab monotherapy specifically, a number of studies have suggested its lack of cost-effectiveness in treating patients with metastatic NSCLC in China (42), the United States (43), the United Kingdom (44), and Singapore (45), although some contradictory results supported its cost-effectiveness in some scenarios, such as in patients with PD-L1 expression ≥50% (43), compared with docetaxel (46), or higher threshold of WTP (47). Regarding the combination of pembrolizumab and platinum-based chemotherapy, several studies have indicated that it represented a cost-effective treatment option for metastatic NSCLC in the United States. However, the same studies have shown that this combination may not be cost-effective in China (48, 49). However, our study suggests that perioperative pembrolizumab (neoadjuvant pembrolizumab plus chemotherapy and adjuvant pembrolizumab monotherapy) has an ICER of $94,222.29 per QALY gained compared with neoadjuvant chemotherapy, with a probability of 97.7% of cost-effectiveness at a WTP threshold at $150,000 per QALY gained. The one-way sensitivity analysis suggested the cost of pembrolizumab as the primary factor in influencing the cost-effectiveness, which is in line with some studies suggesting that the cost of pembrolizumab also has a significant impact on the cost-effectiveness in metastatic NSCLC (48, 49), and our scenario analysis demonstrated that perioperative pembrolizumab stopped being cost-effective when the price of pembrolizumab exceeded $85 per mg. Notably, the price of pembrolizumab fluctuated between $8,817.00 and $10,233.99 per prescription from 2014 to 2021 (50), and the current price of pembrolizumab is around $10,897.12 per prescription (51). Accordingly, if the price of pembrolizumab continues to rise, the cost-effectiveness of perioperative pembrolizumab might be impaired. On the other hand, in scenarios where the WTP threshold is lower, reducing the price of pembrolizumab can be an effective strategy to ensure the treatment remains cost-effective. Additionally, results from our scenario analyses indicated that considering that perioperative pembrolizumab was not cost-effective on a 5-year time horizon, the cost-effectiveness was primarily manifested in its long-term effects.

To our best knowledge, this study offers the first cost-effectiveness analysis on ICI treatment in both neoadjuvant and adjuvant settings. The findings of this study support the cost-effectiveness of perioperative pembrolizumab, despite its high cost, as a viable treatment option for patients with early-stage NSCLC. These results provide valuable insights for healthcare decision-makers. This study also has several limitations. Firstly, the strict inclusion criteria and protocol of the KEYNOTE-671 trial may limit the generalizability of the results to real-world scenarios. For instance, the timeframes for surgery and/or radiotherapy were tightly controlled, with a maximum of 20 weeks from the start of neoadjuvant therapy, and adjuvant therapy had to commence within a specific time window of 4 to 20 weeks after surgery/radiotherapy. Also, the trial population was generally younger and healthier than the general early-stage NSCLC patients, which potentially led to longer survival compared to the real situation. Secondly, a significant number of patients were censored during the follow-up period, which suggests that the fitted survival curves utilized in this study might not precisely reflect the actual survival status of patients with early-stage NSCLC and could lead to overestimation of the treatment effect in both groups. Thirdly, the costs of local recurrence and distant metastases can differ greatly. However, due to a lack of data on patients’ recurrence patterns in KEYNOTE-671, we assumed that patients with disease recurrence would take radiotherapy and chemotherapy, which ineluctably led to overestimated costs for both groups and underestimated cost-effectiveness of perioperative pembrolizumab because patients in the placebo group were more likely to have progressive disease. Finally, we used average sales prices provided by the CMS for our analysis, which can lead to overestimations of drug costs in both treatment groups, because the real-life price concessions given by manufacturers were neglected (52). However, since the key result was primarily sensitive to the cost of pembrolizumab that was positively related to its cost-effectiveness, perioperative pembrolizumab is rather likely to still be cost-effective if the drug costs were lower.

## 5 Conclusions

From the perspective of the United States healthcare payers, perioperative pembrolizumab (neoadjuvant pembrolizumab plus chemotherapy and adjuvant pembrolizumab monotherapy) is a cost-effective treatment for patients with early-stage NSCLC. Sensitivity analyses indicate the strong robustness of this finding and emphasize that the price of pembrolizumab should be the main focus for further enhancing cost-effectiveness.

## Data availability statement

The original contributions presented in the study are included in the article/**Supplementary Material**, further inquiries can be directed to the corresponding author.

## Author contributions

WT: Data curation, Formal Analysis, Methodology, Resources, Software, Visualization, Writing – original draft; LN: Data curation, Formal Analysis, Methodology, Resources, Software, Visualization, Writing – original draft; ZW: Data curation, Writing – review & editing; RL: Data curation, Writing – review & editing; GX: Software, Writing – review & editing; FD: Software, Writing – review & editing; GT: Writing – review & editing; RZ: Conceptualization, Funding acquisition, Project administration, Resources, Writing – review & editing.
