# Supplementary material for: Cost-effectiveness of neoadjuvant pembrolizumab plus chemotherapy with adjuvant pembrolizumab for early-stage non-small cell lung cancer in the United States
Source: Front Immunol. 2023 Sep 26;14:1268070. doi: 10.3389/fimmu.2023.1268070 (PMC10562534; doi:10.3389/fimmu.2023.1268070)
Supplement: Supplementary file 2 [file DataSheet_2.docx]

Supplementary Material

**Table S1.** Summary of AIC and BIC Scores for Parametric Models.

| Scores | Exponential | Weibull | Loglogistic | Lognormal | Gengamma | Gamma | Gompertz |
| --- | --- | --- | --- | --- | --- | --- | --- |
| OS of Pembrolizumab | | | | | | | |
| AIC | 872.04 | 872.15 | 870.72 | 866.59 | 866.74 | 872.51 | 868.81 |
| BIC | 876.03 | 880.11 | 878.69 | 874.55 | 878.70 | 880.48 | 876.78 |
| OS of Placebo | | | | | | | |
| AIC | 1076.88 | 1073.72 | 1074.832 | 1079.22 | 1075.59 | 1073.88 | 1074.26 |
| BIC | 1080.87 | 1081.71 | 1082.81 | 1087.21 | 1087.56 | 1081.87 | 1082.24 |
| EFS of Pembrolizumab | | | | | | | |
| AIC | 1347.44 | 1347.54 | 1338.82 | 1328.47 | 1321.21 | 1348.62 | 1335.67 |
| BIC | 1351.42 | 1355.51 | 1346.79 | 1336.44 | 1333.16 | 1356.59 | 1343.64 |
| EFS of Placebo | | | | | | | |
| AIC | 1749.13 | 1749.42 | 1729.44 | 1721.05 | 1719.50 | 1746.81 | 1748.66 |
| BIC | 1753.13 | 1757.40 | 1737.42 | 1729.03 | 1731.47 | 1754.79 | 1756.64 |

***Abbr.*** *AIC, Akaike information criterion; BIC, Bayesian information criterion; OS, overall survival; EFS, event-free survival.*

**Table S2.** Scenario analyses.

| **Scenarios** | **Treatment** | **Cost, $** | **Incremental Cost, $** | **QALY** | **Incremental QALY** | **ICER ($/QALY)** |
| --- | --- | --- | --- | --- | --- | --- |
| **Time horizons** | | | | | | |
| 5 years | Pembrolizumab | 199424.2 | 105536.2 | 2.61 | 0.33 | 315842.3 |
|  | Placebo | 93888.0 | NA | 2.28 | NA | NA |
| 15 years | Pembrolizumab | 240451.3 | 126310.8 | 5.24 | 2.00 | 63019.22 |
|  | Placebo | 114140.5 | NA | 3.24 | NA | NA |
| 20 years | Pembrolizumab | 250854.4 | 134014.2 | 5.93 | 2.51 | 53379.72 |
|  | Placebo | 116840.3 | NA | 3.42 | NA | NA |
| **Survival distributions** | | | | | | |
| Weibull distribution for both OS estimations | Pembrolizumab | 223699.1 | 113672.8 | 4.04 | 1.07 | 106311.4 |
|  | Placebo | 110026.3 | NA | 2.97 | NA | NA |
| Lognormal distribution for both OS estimations | Pembrolizumab | 224779.1 | 111050.4 | 4.19 | 0.79 | 141379.8 |
|  | Placebo | 113728.7 | NA | 3.40 | NA | NA |
| **Pemetrexed Cost** | | | | | | |
| $1.01 per mg | Pembrolizumab | 207551.9 | 119629.3 | 4.19 | 1.22 | 98226.31 |
|  | Placebo | 87922.6 | NA | 2.97 | NA | NA |
| **Pembrolizumab Cost** | | | | | | |
| $65 per mg | Pembrolizumab | 246050.0 | 136023.7 | 4.19 | 1.22 | 111687.6 |
|  | Placebo | 110026.3 | NA | 2.97 | NA | NA |
| $75 per mg | Pembrolizumab | 266924.3 | 156898.0 | 4.19 | 1.22 | 128827.3 |
|  | Placebo | 110026.3 | NA | 2.97 | NA | NA |
| $85 per mg | Pembrolizumab | 287798.5 | 177772.3 | 4.19 | 1.22 | 145966.9 |
|  | Placebo | 110026.3 | NA | 2.97 | NA | NA |
| $95 per mg | Pembrolizumab | 308672.8 | 198646.5 | 4.19 | 1.22 | 163106.6 |
|  | Placebo | 110026.3 | NA | 2.97 | NA | NA |

***Abbr.*** *OS = overall survival; QALY = quality-adjusted life year; ICER = incremental cost-effectiveness ratio; NA: not applicable.*


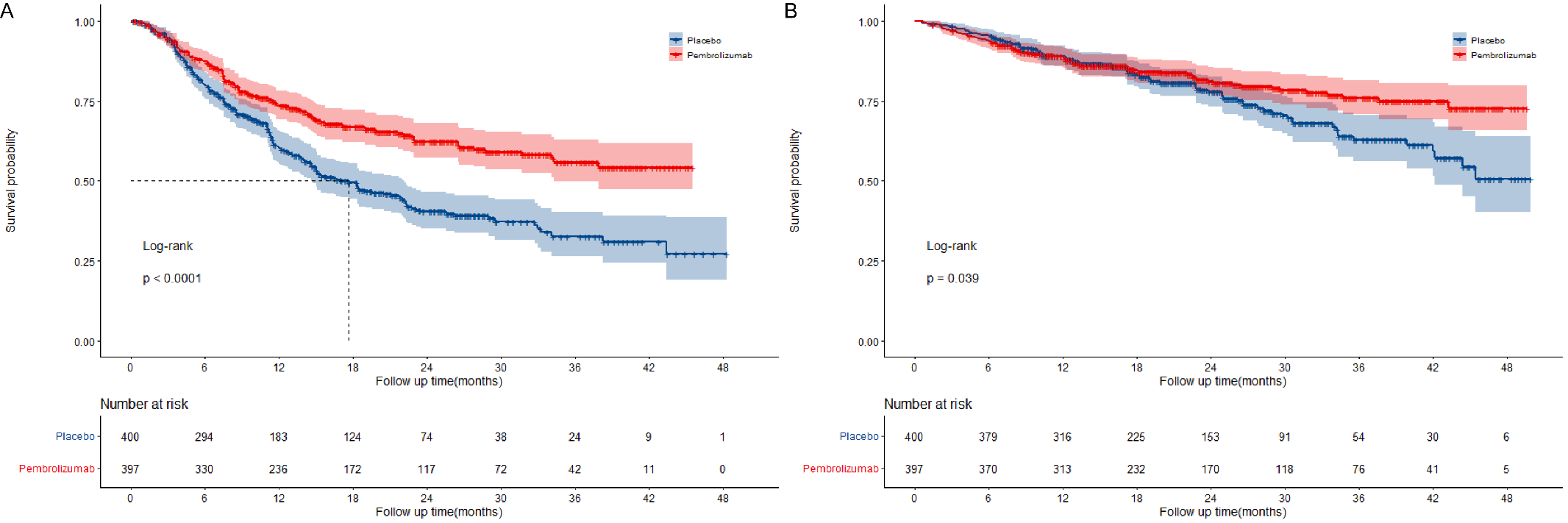


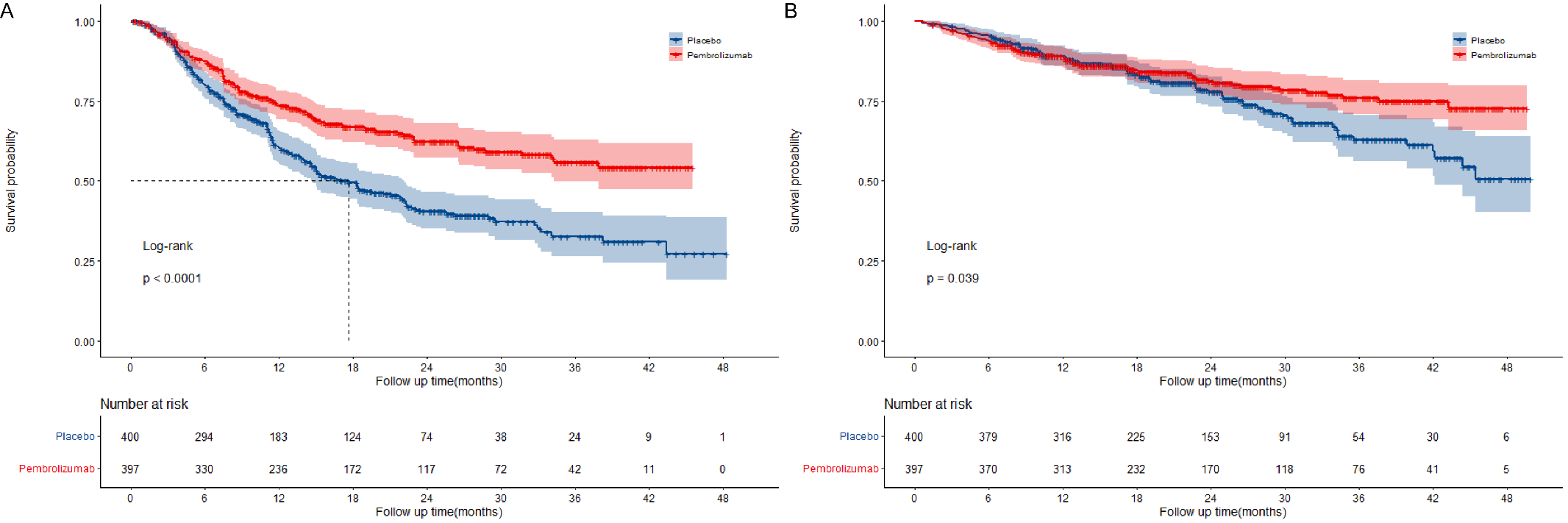


**Figure S1.** Replicated (A) event-free survival and (B) overall survival Kaplan-Meier curves using reconstructed individual patient data.


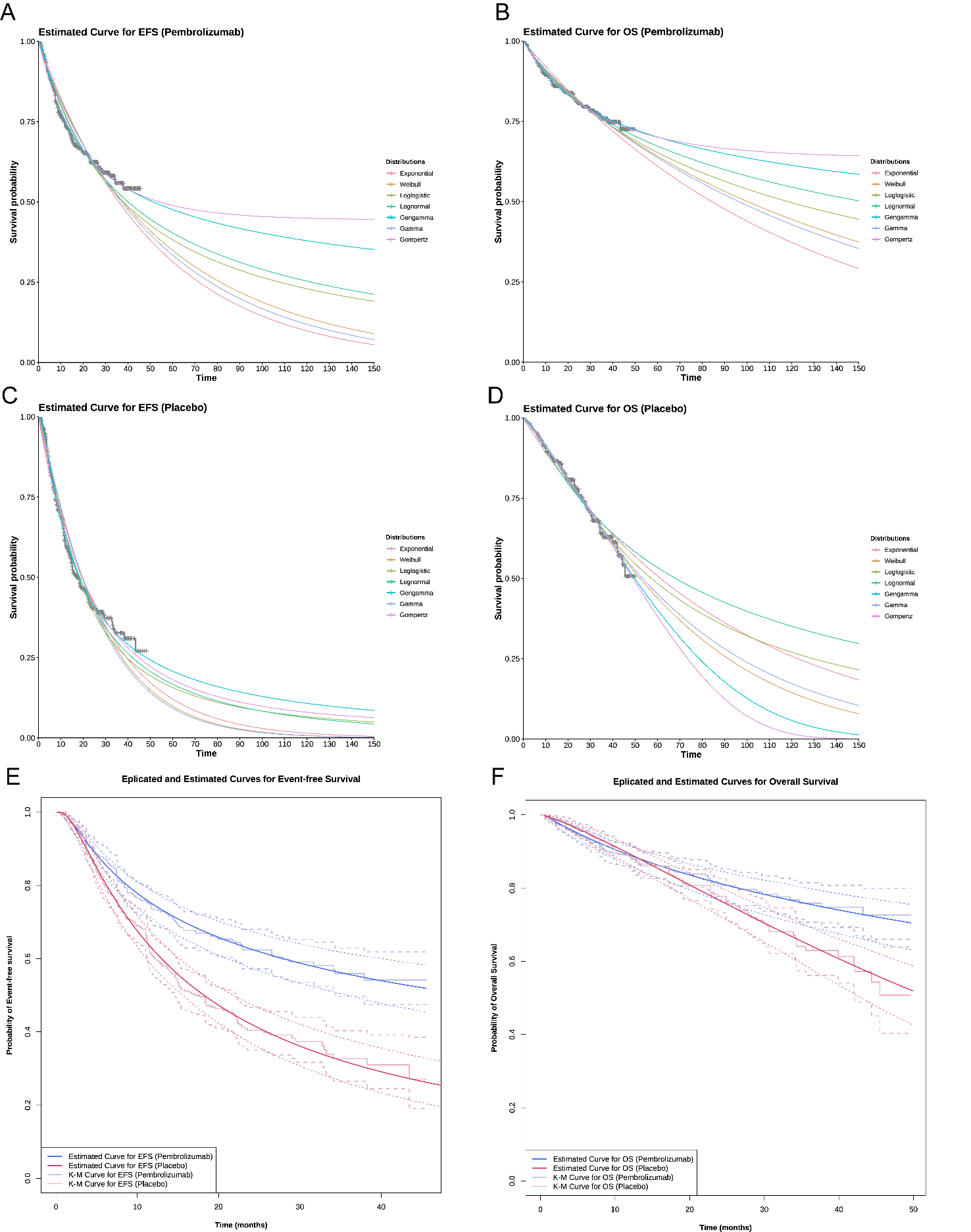


**Figure S2.** Estimated curves for (A) event-free survival and (B) overall survival of the pembrolizumab arm. Estimated curves for (C) event-free survival and (D) overall survival of the placebo arm. (E) Generalized gamma distribution was used to estimate the event-free survival of the two arms. (F) Lognormal and Weibull distribution were used to estimate overall survival of the pembrolizumab arm and the placebo arm, respectively.


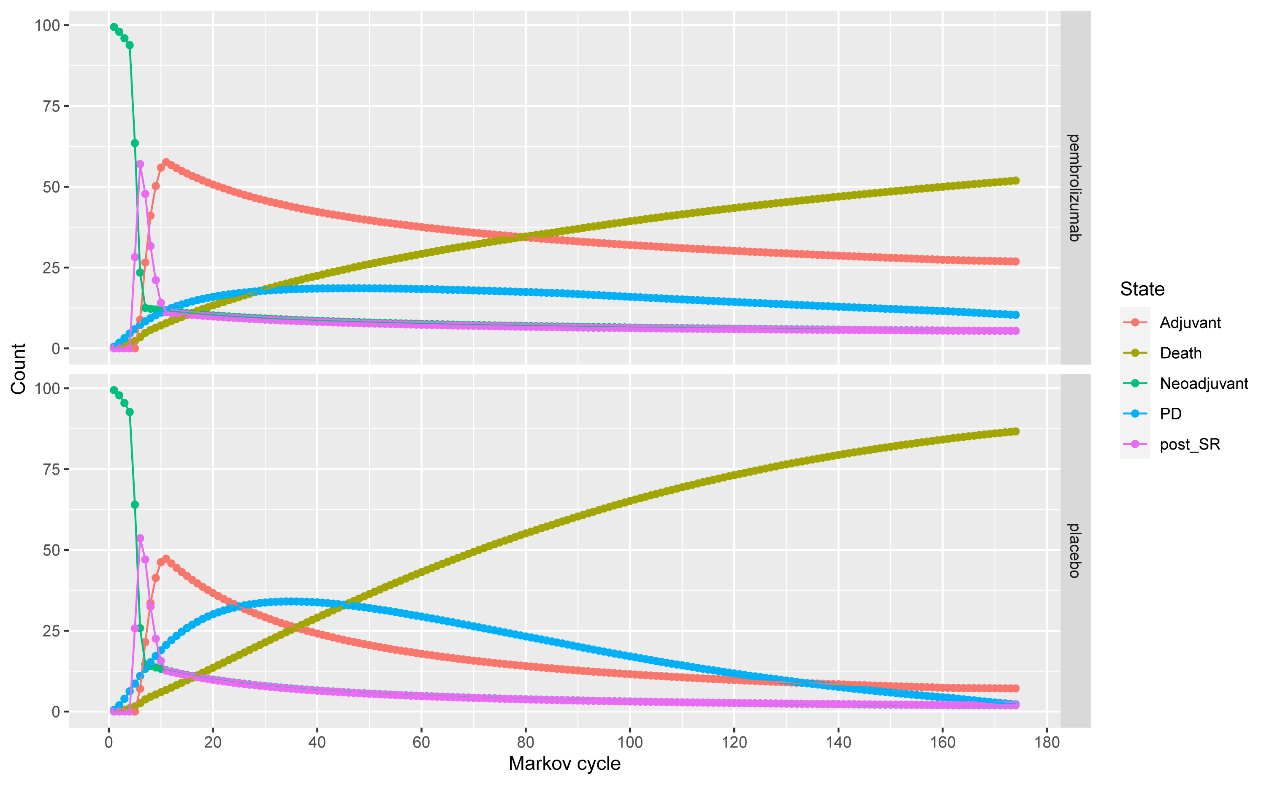


**Figure S3.** Patient counts in 21-day Markov cycles for 10 years (174 cycles).


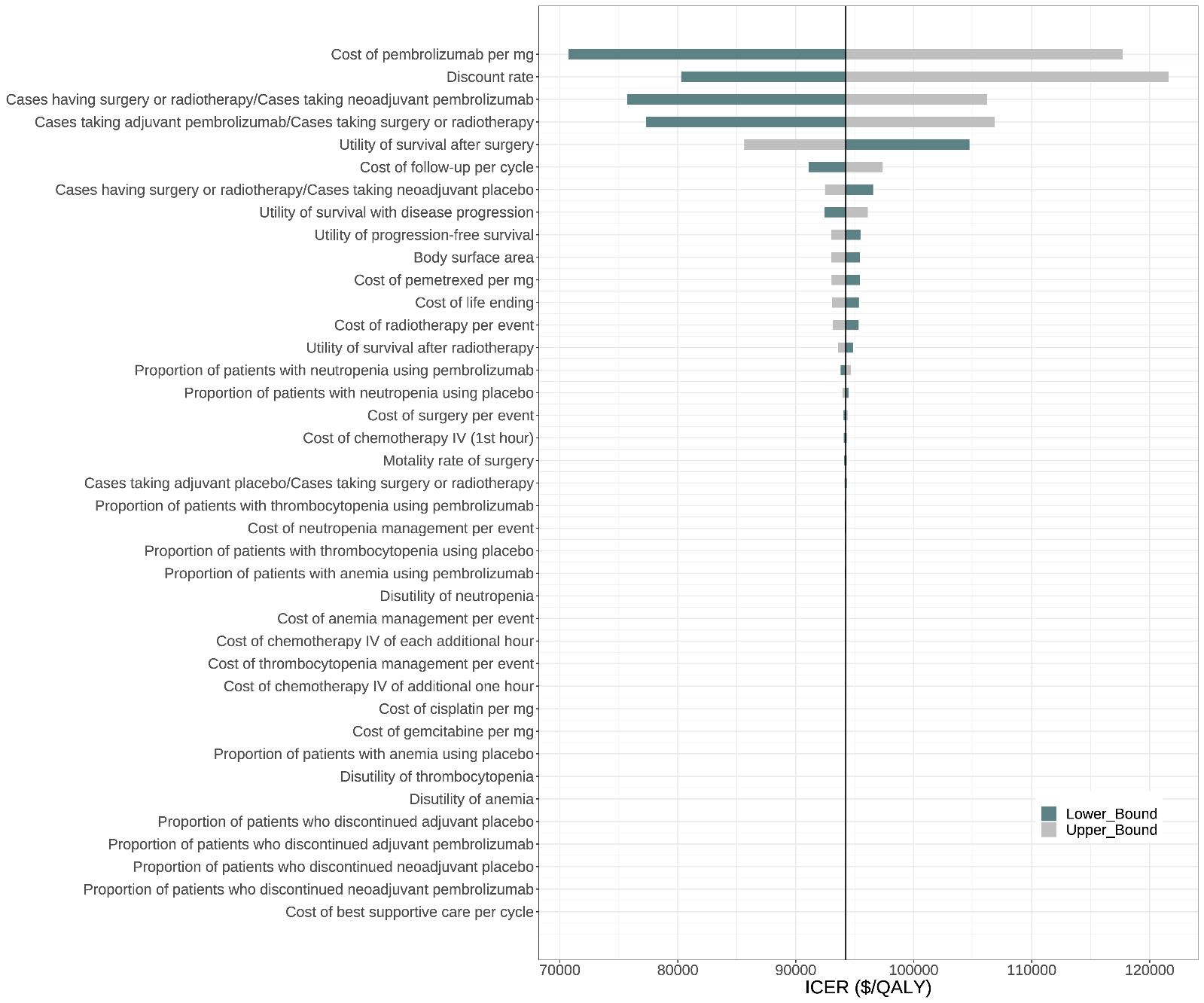


**Figure S4.** Full tornado diagram of all the parameters included in one-way sensitivity analysis.


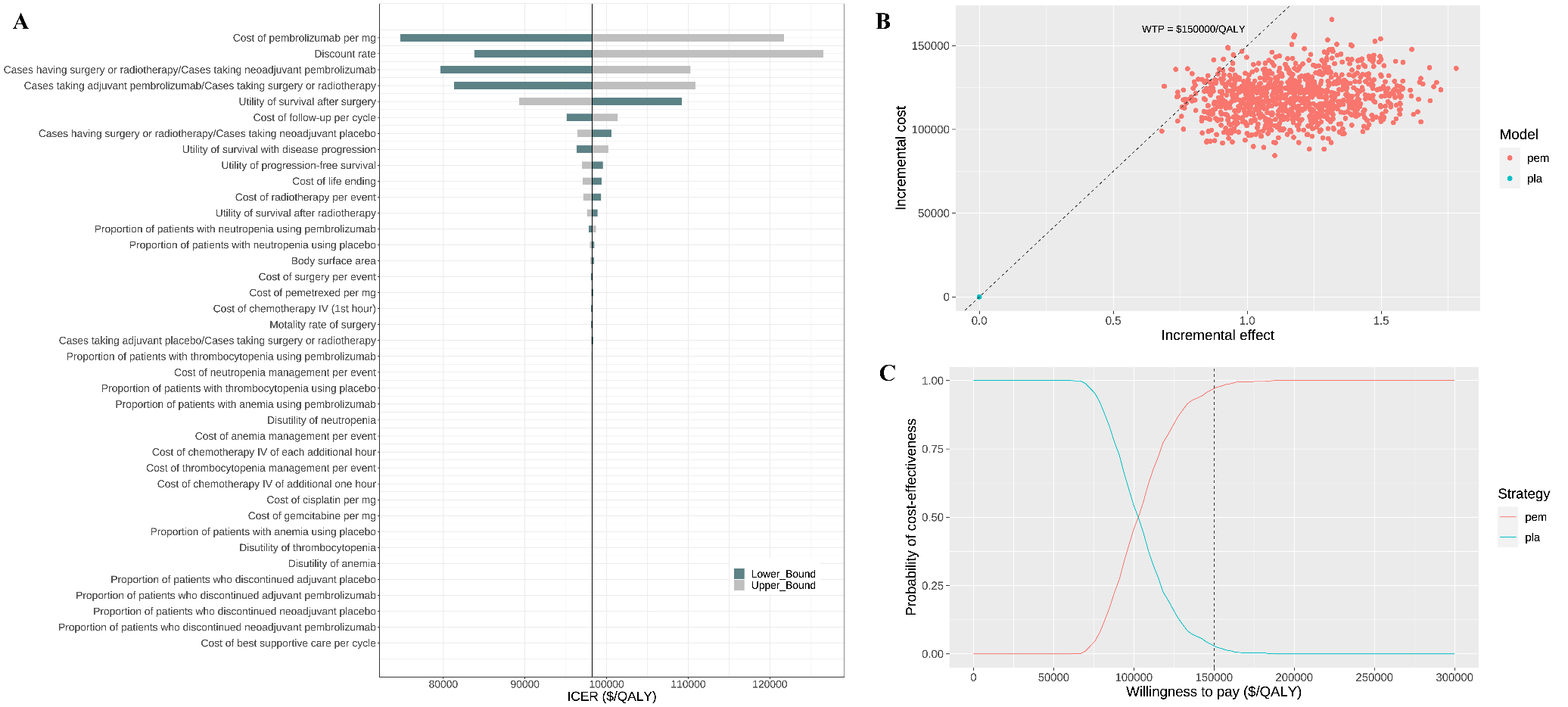


**Figure S5.** Sensitivity analyses of the scenario analysis using varied prices of pemetrexed (from $0.79 per mg to $1.23 per mg). A) One-way sensitivity analysis; B) Incremental cost ($) and incremental effect (QALY) incurred by 1,000 probabilistic resamplings int the probabilistic sensitivity analysis; C) Probability of cost-effectiveness at varying willingness-to-pay. The dashed line represents the willing-to-pay threshold of $150,000 per QALY gained. ***Abbr.*** *pem = pembrolizumab; pla = placebo.*
